# Diverse Bacteria Utilize Alginate Within the Microbiome of the Giant Kelp *Macrocystis pyrifera*

**DOI:** 10.3389/fmicb.2018.01914

**Published:** 2018-08-20

**Authors:** Jordan D. Lin, Matthew A. Lemay, Laura W. Parfrey

**Affiliations:** ^1^Department of Botany, Biodiversity Research Centre, The University of British Columbia, Vancouver, BC, Canada; ^2^Hakai Institute, Heriot Bay, BC, Canada; ^3^Department of Zoology, University of British Columbia, Vancouver, BC, Canada

**Keywords:** kelp, microbiome, metagenomics, *Macrocystis*, alginate, epibiota

## Abstract

Bacteria are integral to marine carbon cycling. They transfer organic carbon to higher trophic levels and remineralise it into inorganic forms. Kelp forests are among the most productive ecosystems within the global oceans, yet the diversity and metabolic capacity of bacteria that transform kelp carbon is poorly understood. Here, we use 16S amplicon and metagenomic shotgun sequencing to survey bacterial communities associated with the surfaces of the giant kelp *Macrocystis pyrifera* and assess the capacity of these bacteria for carbohydrate metabolism. We find that *Macrocystis*-associated communities are distinct from the water column, and that they become more diverse and shift in composition with blade depth, which is a proxy for tissue age. These patterns are also observed in metagenomic functional profiles, though the broader functional groups—carbohydrate active enzyme families—are largely consistent across samples and depths. Additionally, we assayed more than 250 isolates cultured from *Macrocystis* blades and the surrounding water column for the ability to utilize alginate, the primary polysaccharide in *Macrocystis* tissue. The majority of cultured bacteria (66%) demonstrated this capacity; we find that alginate utilization is patchily distributed across diverse genera in the Bacteroidetes and Proteobacteria, yet can also vary between isolates with identical 16S rRNA sequences. The genes encoding enzymes involved in alginate metabolism were detected in metagenomic data across taxonomically diverse bacterial communities, further indicating this capacity is likely widespread amongst bacteria in kelp forests. Overall, the *M. pyrifera* epibiota shifts across a depth gradient, demonstrating a connection between bacterial assemblage and host tissue state.

## Introduction

Heterotrophic bacteria play an essential role in the world’s oceans by metabolizing and remineralising dissolved and particulate organic carbon that would otherwise be unavailable to higher trophic levels (Azam and Malfatti, 2007). These interactions provide a key contribution to biogeochemical cycling by liberating refractory carbon, shuttling carbon between coastal systems (Säwström et al., 2016), and reducing the sequestration of carbon to the deep ocean (Jiao et al., 2010).

Within the global oceans, kelp forests are one of the most productive ecosystems and a large reservoir of organic carbon (Reed et al., 2008), contributing an estimated 5662 g C m^-2^ yr^-1^ to global net primary production (Krumhansl and Scheibling, 2012). The majority of kelp carbon is released to the environment as detritus (i.e., particulate organic carbon; Krumhansl and Scheibling, 2012), while a smaller portion is exuded as dissolved organic matter (Reed et al., 2015). Both of these substrates represent substantial pools of organic carbon for bacterial utilization. Given the abundance of carbon within kelp forest ecosystems and the interdependence between bacterial metabolism and marine productivity, the kelp forest is an important system in which to study microbial carbon cycling and oceanic nutrient fluxes.

The giant kelp *Macrocystis pyrifera* is the largest, fastest growing macroalgal species (Ravanal et al., 2017) and serves as the structural foundation in Pacific kelp forests (Steneck et al., 2002), yet the diversity and metabolic capacity of bacteria that cycle *Macrocystis* carbon is poorly known. The epibiota of *Macrocystis* (Michelou et al., 2013), and kelp generally (Lemay et al., 2018), are distinct from that of the water column. Yet, kelp influence the water column microbiota (Lam and Harder, 2007; Chen and Parfrey, 2018) and their carbohydrate degradation capacity (Clasen and Shurin, 2015). Studies comparing across seaweeds find species-specificity of associated bacteria in taxonomic profiles (Lachnit et al., 2009; Lemay et al., 2018) and functional profiles, with functional differences in part reflecting metabolism of host polysaccharides (Roth-Schulze et al., 2016). Across kelps, host tissue age (Bengtsson et al., 2012) and condition (Marzinelli et al., 2015) shape epibiota diversity and composition. For example, Bengtsson et al. (2012) documented seasonal microbiota succession on blades of *Laminaria hyperborea*, and Lemay et al. (2018) show an analogous pattern in which perennial and annual kelp species harbor different epibiotic communities.

Bacteria transform algal-derived carbon by utilizing a diverse suite of degradative enzymes (Wang et al., 2016) that target numerous host polysaccharides (Dong et al., 2012; Martin et al., 2015). Brown algae, including kelp, produce an extracellular matrix primarily composed of the polysaccharides alginate, cellulose, and fucoidan (Michel et al., 2010), with the relative abundance of these polysaccharides varying across species and seasons. Alginate is the dominant polysaccharide in several kelp species, including *M. pyrifera*, where it comprises roughly 15–25% of dry weight, with highest concentrations observed in the fall (Whyte and Englar, 1978) and in young blades (McKee et al., 1992). Polymeric alginate is metabolized via alginate and oligoalginate lyase enzymes that are typically found within bacterial operons (Thomas et al., 2012). Alginate assimilation is initiated by the depolymerization of polymeric alginate, which is typically catalyzed by extracellular alginate lyases, although direct uptake of the polymer has been reported (Hisano et al., 1995). Oligomeric products are then imported into the bacterial periplasm, where they are further degraded into monosaccharides by oligoalginate lyases (Thomas et al., 2012) prior to transport into the bacterial cytosol. Because alginate is one of the primary organic carbon sources in kelp forests and a known substrate of bacterial metabolism, its utilization by bacteria is likely an important process in coastal carbon turnover.

The objective of this study is to determine the distribution and variability of alginate utilization among bacteria in Pacific *Macrocystis* forests, and to reconstruct the broader communities in which this activity occurs. We perform amplicon and shotgun metagenomic sequencing on epibiotic and water column communities to reconstruct their taxonomic and carbohydrate metabolic profiles, and provide the first metagenomic survey of alginate metabolic genes in kelp forests. We also assay cultured bacteria isolated from the kelp forest to determine their ability to degrade polymeric alginate and metabolize it for growth (referred to together as alginate utilization). These data provide a better understanding of the relationship between taxonomic and functional diversity in marine bacterial communities.

## Materials and Methods

### Surface-Associated Microbial Communities

*Macrocystis pyrifera* blades were sampled by scuba in August, 2015 from three subtidal kelp beds adjacent to Calvert Island, British Columbia (Site 1 (Stryker): 52 04.962 N, 128 21.883 W; Site 2 (Triquet): 51 48.343 N, 128 15.479 W; Site 3 (Goose South): 51 55.226 N, 128 27.571 W; **Supplementary Data Sheet S2**). These sites were chosen due to their similar oceanographic properties, depths (Site 1 – 5 m; Site 2 – 5 m; Site 3 – 7 m), and inclusion in long term monitoring projects. At each site, five kelp blade replicates were sampled at the bottom (1 m above sediment), middle (1 m below surface), and top (surface) of the water column. Each blade was removed and brought to the surface in a sterile Ziploc^®^ bag. On the surface, each blade (*n* = 45) was rinsed with sterile seawater for 10 s to remove transient microbes, and then a 10 cm^2^ area in the center of the blade was swabbed with a sterile Puritan^®^ cotton swab for 10 s. Swabs were immediately transferred into sterile cryovials (VWR) and placed on ice for transport back to the lab, where they were stored at -80°C. Water samples from each site were collected from the same depths as kelp blades and were stored in sterile 500 ml Nalgene^®^ bottles (*n* = 12). Water was pre-filtered at 150 μm to remove larger organisms and bacteria were subsequently filtered from the water samples using a Cole-Parmer MasterFlex L/S peristaltic pump with a 0.22 μm Durapore^®^ membrane filter (Merck Millipore Ltd.). Filters from each water sample were immediately stored at -80°C in individual Whirl-Pak^®^ bags.

Bacterial DNA from the kelp blade swabs and water column filters was extracted with the MoBio PowerSoil^®^-htp 96 well DNA extraction kit using standard protocols (Mo Bio Laboratories, United States). The V4 region of the 16S rRNA gene was amplified by PCR using redesigned versions of the primers *515f/806r* (Caporaso et al., 2012): *515f*: 5′–GTGYCAGCMGCCGCGGTAA–3′, *806r*: 5′–GGACTACNVGGGTWTCTAAT–3′. These primers have been modified to include a 12 bp Golay barcode on the forward primer and degeneracies were added to improve taxonomic coverage^1^. Each PCR consisted of 10 μl of 5-Prime Master Mix, 1 μl of each primer (final concentration = 0.2 μM), 2 μl of DNA, and PCR grade water to a final volume of 25 μl. The PCR protocol consisted of an initial denaturation step at 94°C for 3 min, followed by 25 cycles of denaturation at 94°C for 45 s, primer annealing at 50°C for 60 s, and extension at 72°C for 90 s, with a final extension step of 72°C for 10 min. PCR products were quantified using Quant-IT Pico Green^®^ ds DNA Assay Kit (Life Technologies). Sample DNA was then pooled in equal amounts (25 ng) and purified using the MoBio UltraClean^®^ PCR clean-up kit. Quantitation of the pooled library and paired-end Illumina MiSeq sequencing (2 × 300 bp) were performed at the Integrated Microbiome Resource facility at Dalhousie University (Halifax, Canada).

The resulting raw sequencing reads were demultiplexed in QIIME v1.9.1 (Caporaso et al., 2010b) using split_libraries_fastq.py, which yielded an average of 86,857 reads/sample. Demultiplexed reads were trimmed to 250 bp using the FastX Toolkit^2^ and clustered using Minimum Entropy Decomposition (MED; Eren et al., 2015) into nodes analogous to operational taxonomic units (OTUs). This clustering was performed as implemented in the Oligotyping microbial analysis software package (Eren et al., 2013) and carried out with the minimum substantive abundance parameter (-M) set at 250 reads. All other parameters retained the default settings. MED OTUs were taxonomically identified using UCLUST (Edgar, 2010) and assign_taxonomy.py in QIIME by querying the SILVA SSU Ref NR 128 database (**Supplementary Data Sheet S4**). OTUs were subsequently filtered for host contamination by removing chloroplast and mitochondrial annotations, and by sample occurrence (≥2 samples) and read count (≥100 reads per OTU). The remaining OTU representative sequences were aligned with PyNAST v.1.2.2 (Caporaso et al., 2010a) using the SILVA SSU Ref NR 128 alignment as a template and a tree was constructed in QIIME using FastTree (Price et al., 2010). An OTU heatmap was constructed in the R statistical environment (all analyses in R performed with v3.4.0; R Core Team, 2017).

### Bacterial Community Comparisons

To quantify the richness of culture-independent bacterial communities, the Chao1 metric (Chao, 1984) was calculated for each sample in QIIME using alpha_diversity.py. Samples were first rarefied at a depth of 30,000 sequences. Richness was compared using mixed effect analysis of variance (ANOVA) with the lmerTest package (Kuznetsova et al., 2015) in R. Briefly, when comparing *Macrocystis* and water samples, site was set as a random effect while depth (bottom, middle, surface) and sample type (seawater, macroalgae) were both set as fixed effects. When comparing only kelp blades, site was again set as a random effect and depth was set as a fixed effect. Pairwise comparisons of kelp communities across depths were performed within lmerTest as Student’s *t*-tests.

We compared bacterial community composition using the weighted UniFrac metric, which takes abundance into account (Lozupone et al., 2007). Beta diversity distance matrices were generated in QIIME at a rarefaction depth of 30,000 sequences/sample and visualized as Principal Coordinates plots in R. Communities were compared using a permutational multivariate analysis of variance (PERMANOVA; Anderson et al., 2005) and a permutational analysis of within group multivariate dispersion (PERMDISP; Anderson, 2004), both with 9999 permutations and implemented in Primer (v.6; Clarke and Gorley, 2006). When comparing all bacterial communities, both depth and sample type were set as fixed effects while site was set as a random effect. For comparisons of *Macrocystis* samples only, depth was once again a fixed effect and site a random effect. Pairwise PERMANOVA analyses conducted within Primer were used to compare *Macrocystis* communities across depths.

Testing for the differential abundance of bacterial genera across *Macrocystis* blade depths was performed in R using the two-tailed Welch’s *t*-test (t.test). Benjamini–Hochberg adjusted *p*-values were used to account for multiple hypothesis testing (Benjamini and Hochberg, 1995). Read counts were normalized to total sample counts prior to analysis. Genera were considered differentially abundant if their FDR corrected *p*-value < 0.1 and log2 fold change across depths was ≥| 2| .

### Carbohydrate Metabolic Profile Reconstruction

To assess the capacity for alginate utilization in kelp forest bacterial communities, a subset of samples were sent for shotgun metagenomic sequencing using the same DNA as 16S rRNA gene sequencing. These samples consisted of 10 kelp (5 bottom depth, 5 middle depth) and 5 water samples (4 bottom depth, 1 surface depth) from one kelp bed (Site 3). Metagenomic libraries were prepared using the Illumina Nextera XT kit and 150-bp fragments and sequenced on an Illumina NextSeq 550 at the Integrated Microbiome Resource facility at Dalhousie University. Quality statistic generation, paired-end read merging, and quality filtering were performed with VSEARCH (Rognes et al., 2016) on the resulting raw sequencing reads. Merging was performed with default parameters (except maximum mismatch was reduced to 2 nucleotides) and non-merged forward reads were added to the merged reads to increase coverage. Reads were trimmed at the beginning to remove low quality bases, quality filtered (minimum length: 100-bp; maximum error rate: 0.005), and truncated once base quality dropped below 30. This yielded 7,924,598 reads with an average length of 147-bp. The mean sequences per sample was 528,307, and one kelp sample with 1169 reads was removed prior to analysis due to low sequence count.

Open reading frames (ORFs) were predicted in MetaPathways v2.5.1 (Konwar et al., 2015) using the prodigal algorithm and a minimum peptide length of 33. Predicted ORFs were annotated in MetaPathways with the LAST algorithm and default run parameters against the Carbohydrate Active Enzymes database (CAZy; release 2014.09.04; Lombard et al., 2014). This resulted in 6,799,723 ORFs, of which 138,785 were annotated to the CAZy database (**Supplementary Data Sheets S5**, **S6**). Subsequent filtering to remove eukaryotic, viral, archaeal, and unclassified proteins resulted in 126,084 ORFs corresponding to 26,509 CAZy annotations (**Supplementary Data Sheets S7**, **S8**).

Carbohydrate Active Enzymes functional profiles were rarefied at a depth of 1000 sequences/sample prior to comparisons of composition and richness. Profile dissimilarity matrices were generated using the Bray-Curtis metric (Bray and Curtis, 1957) and differences in composition were tested using PERMANOVA and PERMDISP in Primer v.6 (9999 permutations each; *p* < 0.05), and visualized with principle coordinate plots. Functional richness was determined by the observed number of enzyme annotations and families within each sample and compared using the two-tailed Welch’s *t*-test in *R* (t.test). Communities were compared across sample type and depth separately, instead of a mixed effect design, as the water samples did not sufficiently represent multiple depths. Testing for an association between functional and taxonomic richness was performed using the two-tailed Spearman’s rank correlation test in *R* (cor.test).

Differential abundance analysis for CAZy families and individual gene annotations was performed using the two-tailed Welch’s *t*-test in *R*. Genes with <100 total reads and families with <1000 total reads were excluded from the analysis in order to compare only high abundance groups. Read counts were normalized to sample counts and p-values were adjusted for multiple hypothesis testing using the Benjamini–Hochberg method (Benjamini and Hochberg, 1995). Genes and families were considered differentially abundant if their FDR corrected *p*-value < 0.1 and log2 fold change ∼| 1| or greater. The log2 fold change threshold was relaxed relative to genus enrichment due to low sequence coverage in the metagenomic dataset.

To determine how well the metagenomic dataset corresponded to the 16S amplicon data, and to better link the two bacterial community profiles, we performed closed reference OTU picking in QIIME (pick_closed_reference_otus.py) on the quality filtered metagenomic sequences against the SILVA SSU Ref NR 128 database. OTUs were taxonomically assigned using UCLUST (Edgar, 2010) and assign_taxonomy.py, and OTUs assigned to chloroplast or mitochondria were removed. Taxa summary plots were then constructed for comparison with the 16S rRNA gene amplicon dataset.

### Cultured Bacterial Isolates and Detection of Alginate Utilizing Bacteria

Bacteria were cultured from a subset of the macroalgal blades and water column samples used for amplicon sequencing (**Supplementary Data Sheet S3**). *Macrocystis* blades from the bottom and middle depth at each site that were swabbed for sequencing were immediately swabbed again for culturing. These swabs were plated directly onto both alginate and tryptone marine agar media (*n* = 30 for each media type). Alginate marine agar media was made using a previously published protocol (Clasen and Shurin, 2015) and consisted of distilled water, Instant Ocean^®^ (to salinity∼31 ppt), 1.5% agar, and 0.3% sodium alginate (Sigma-Aldrich A0682). Tryptone marine agar media was made using distilled water, Instant Ocean^®^ (to salinity∼31 ppt), 1.5% agar, and 1% tryptone (BD REF211705). Liquid versions of both media types were prepared in identical fashion with agar excluded. One water sample from mid and bottom depths at each site was also plated on each media type (*n* = 12). Plates were sealed with parafilm and stored at 4°C for 2 weeks to facilitate transport from the remote field site to the lab. Once in the lab cultures were incubated at 12°C, as this was approximately the *in situ* water temperature when sampling. After plates had been incubated for several days, 5–10 colonies displaying morphological variation were isolated using standard microbiological techniques. Subsequent assays and maintenance of cultures all occurred at 12°C.

### Growth With Alginate as the Sole Carbon Source

Isolated bacteria were inoculated in triplicate into 96 well microplates containing alginate marine media to determine if any were capable of utilizing alginate as a sole carbon source. Growth was measured daily using turbidimetry over 4 days. Isolates were considered to be growing if absorbance readings at the first and final time point were significantly different (two-tailed paired *t*-test; *p* < 0.05), a minimum increase of 0.1 OD_492_ was detected, and mean absorbance increased by ≥50%.

### Alginate Utilization Assays

We aimed to broadly detect alginate utilization by bacteria and performed two distinct assays on each isolate to better capture the different stages of the alginate metabolic pathway. The use of two assays allowed us to partially account for the complex metabolic interactions and substrate partitioning that have been reported for alginate (Wong et al., 2000; Kita et al., 2016). For example, some bacteria that do not degrade polymeric alginate can metabolize alginate oligosaccharides (Hehemann et al., 2016) and some taxa can directly uptake the polymer (Hisano et al., 1995). One assay employed here assessed extracellular degradation of polymeric alginate and another tested for enhanced growth yielded by alginate metabolism within the cell. It should be noted these assays were chosen to detect possible interactions with extracellular, kelp-derived alginate, but they do not distinguish this from the biosynthesis of alginate, of which some bacteria are capable and similar enzymes are involved (Kim et al., 2011). Furthermore, quantitative assays were chosen over traditional plate staining methods to minimize subjectivity, due to the lack of substrate specificity reported in analogous assays of polysaccharide degradation (Meddeb-Mouelhi et al., 2014), and to provide consistent and conservative thresholds for detecting alginate utilization.

Metabolism of alginate for growth was assessed by comparing the growth of liquid cultures with and without alginate supplemented to the medium. The alginate supplemented media consisted of tryptone marine media (see above) with 0.3% sodium alginate added. Each isolate was grown for 3 days prior to the experiment. This likely corresponded to post-exponential phase for most isolates, but we did not construct individual growth curves. Cultures were then inoculated in triplicate into a 96 well plate containing tryptone media and a plate containing alginate supplemented media. Growth was then measured daily using turbidimetry over 4 days. Isolates were considered to be metabolizing alginate if they grew in alginate supplemented media (according to the criteria listed above for the alginate only media) and net growth in alginate supplemented media was 50% greater than in un-supplemented media. It should be noted that while this assay was used to detect increased growth in alginate supplemented media, it is unable to identify organisms who utilize alginate but achieve the same maximal growth in culture, or taxa whose metabolism of tryptone is inhibitory to the metabolism of alginate.

The second aspect of alginate utilization, degradation of polymeric alginate, was assessed by measuring the strong absorption of UV light characteristic of de-polymerized alginate, due to the formation of C-4 = C-5 double bonds within alginate oligosaccharides (Wong et al., 2000; Han et al., 2016). As above, each isolate was grown for 3 days prior to the experiment and inoculated in triplicate into a 96 well plate containing alginate marine media. UV absorbance was measured daily over a 4 days time period. Isolates were considered to degrade alginate if the absorbance readings at the first and final time point were significantly different (paired *t*-test, *p* < 0.05), a minimum change of 0.1 OD_230_ was detected, and absorbance increased by ≥50%. This assay was used to detect the increase in UV absorbance over the course of the growth period, however, it was not used to detect an initial increase in absorbance followed by a reduction (owing to the uptake of alginate oligosaccharides). Thus, bacterial isolates that rapidly imported the cleaved alginate oligosaccharides at a rate greater than depolymerization were not detected using this technique. Negative controls for all assays consisted of media without bacterial inoculum.

### Isolate Sequencing and Taxonomy Assignment

Isolates assayed for alginate utilization were sequenced at the V4–V9 region of the 16S rRNA gene. DNA was extracted using Prepman Ultra^®^ and amplified with primers *515f/1492r*: *515f*: 5′–GTGYCAGCMGCCGCGGTAA–3′, *1492r*: 5′–CGGTTACCTTGTTACGACTT–3′. Each PCR contained 12.5 μl of 2X Phusion Flash Master Mix^®^, 1.25 μl of each primer (final concentration: 0.5 μM), 1 μl of DNA, and PCR grade water to final volume of 25 μl. PCR was performed with an initial denaturation temperature of 98°C for 10 s, followed by 30 cycles of denaturation at 98°C for 1 s, annealing at 52.3°C for 5 s, and extension at 72°C for 15 s, with final extension at 72°C for 1 min. PCR products were Sanger sequenced at the Genome Quebec Innovation Centre at McGill University (Montreal, Canada).

Isolate sequence data was initially trimmed in Geneious (v.9.1; Biomatters) to remove low quality ends and ambiguities. The resulting sequences were then aligned using the SILVA Incremental Aligner (SINA v1.2.11; Pruesse et al., 2012), queried against the SILVA SSU Ref 128 database (minimum identity 95%; Quast et al., 2012), and the highest matching taxon was recorded. Sequences were subsequently queried against the SILVA SSU Ref 128 database locally using BLAST+ (blastn; Camacho et al., 2009) to confirm taxonomy and retrieve additional quality scoring metrics. Isolate sequences were then collapsed into OTUs in QIIME by clustering at 100% sequence identity using UCLUST (Edgar, 2010) and a representative sequence was picked for each OTU. For each 100% OTU, we then aligned all sequences that were collapsed into the OTU with MUSCLE (Edgar, 2004) to confirm that sequences were 99.8–100% identical; they were.

Phylogenetic trees of isolate OTUs and reference sequences were constructed using RAxML v.8.2.9 (Stamatakis, 2014) for the three major bacterial clades detected. Briefly, OTU representative sequences were aligned to the SILVA SSU Ref NR 128 alignment using PyNAST within QIIME. Aligned sequences, along with a filtered SILVA SSU Ref NR 128 alignment and tree, were then used to construct a Maximum Likelihood tree using the GAMMA model of rate heterogeneity and the evolutionary placement algorithm (Berger et al., 2011). The SILVA alignment and tree were filtered to retain only genera detected in either the culture-independent or cultured communities of *Macrocystis* and the water column for each clade. Reference sequences for each genus were selected because they were either the type species or a common marine taxon. The number of reference sequences to include for each genus was chosen relative to the number of OTUs falling within that genus. Annotation of phylogenetic trees was performed using the ggtree package (Yu et al., 2017) in R.

We assessed the taxonomic composition of the cultured community with high-throughput 16S rRNA gene sequencing by scraping all colonies off of a subset of culture plates with a sterile cell scraper following isolation of individual colonies; 1 sample per plate. These pooled bacterial colonies were then processed with the same DNA extraction, PCR, and amplicon sequencing procedures as the uncultured communities (above). The plates used here were from one water and three kelp samples from each site, and both media types were included (*n* = 24 total; water = 6, middle depth kelp blades = 18).

### Figure Generation

All figures were generated and exported in the R statistical environment (v3.4.0; R Core Team, 2017). Minor modifications, when needed, were performed using Inkscape v0.91.

### Accession Numbers

Raw Illumina MiSeq and NextSeq reads, along with associated MiMARKS compliant metadata, have been accessioned in the European Bioinformatics Institute^3^ (Study Accession Number: PRJEB21672). Raw Sanger sequencing reads have been accessioned in GenBank^4^ (Accession Numbers: MF443456 – MF443740).

## Results

### Bacterial Communities on *Macrocystis pyrifera* and in the Surrounding Water Column

**FIGURE 1:**
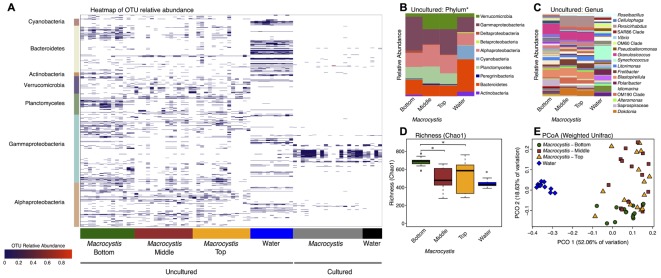
Bacterial diversity associated with *Macrocystis* blades and the nearby water column. **(A)** Heatmap of the relative abundance of operational taxonomic units (OTUs) detected on *Macrocystis* blades from different depths and in the water column from cultured and uncultured samples. OTUs are ordered by taxonomy and the major bacterial clades detected are indicated on the *y*-axis. **(B–E)** Uncultured samples only. **(B)** Taxa summaries of uncultured communities on *Macrocystis* and in the water column at the Phylum level (^∗^Class for Proteobacteria). All samples within a sample type merged. **(C)** Genus level plot of **(B)**. **(D)** Bacterial species richness (Chao1 index) is higher on the *Macrocystis* surface compared to the water column (*N* = 11), and greater on blades at bottom depth (*N* = 14) compared to middle (*N* = 15) and top (*N* = 15) depths. Statistical tests summarized in text; ^∗^*p* < 0.01. **(E)** Principle coordinate plot of Weighted UniFrac distance for kelp and water bacterial communities.

We sampled the cultured and uncultured epibiota on *M. pyrifera* blades from three depths, along with 11 nearby water column communities, within three kelp beds adjacent to Calvert Island, British Columbia. Overall, these bacterial assemblages are composed of 1001 OTUs assigned to 198 genera. Unsurprisingly, there are substantial differences between cultured and uncultured communities and fewer OTUs detected in cultured communities (**Figure 1A**). At broad taxonomic levels, uncultured communities are primarily composed of Gammaproteobacteria, Alphaproteobacteria, Verrucomicrobia, Planctomycetes, and Bacteroidetes (**Figures 1A,B**), while Gammaproteobacteria and Bacteroidetes dominate cultured communities (**Figure 1A** and **Supplementary Figure S1**). The uncultured *M. pyrifera* epibiota differs strongly from the uncultured bacterial community found in the water column; while many OTUs are shared across these sample types their relative abundance differs markedly (**Figure 1A**). Differences between water and *M. pyrifera* are driven in part by high abundance of Cyanobacteria (mostly *Synechococcus*) in the water, but near absence on *M. pyrifera*, and abundant Verrucomicrobia (*Persicirhabdus*) and Planctomycetes (*Blastopirellula*) on *M. pyrifera* blades from middle and top depths (**Figures 1A,B**). Within shared phyla we also observe turnover at the genus level across Proteobacteria and Bacteroidetes (**Figure 1C**). The cultured community is quite similar between the water column and *M. pyrifera*, likely due to the selectivity of culturing (**Figure 1A** and **Supplementary Figure S1**).

At higher resolution, bacterial diversity again differs between the *M. pyrifera* epibiota and surrounding water column across uncultured communities, and also across blade depth for *M. pyrifera*. Unless otherwise specified we focus on the uncultured bacterial communities from this point on. We used a mixed effect ANOVA implemented in lmer to test for differences in richness, as measured by the Chao1 index, between sample types (water versus *Macrocystis* blades; fixed effect) and across *Macrocystis* blade depth (top, middle, bottom; fixed effect), while accounting for site, which was included as a random effect. Richness differs significantly across sample type (Chao1; *F* = 18.2, df = 1, *p* = 0.0001) and blade depth (*F* = 9.7, df = 2, *p* = 0.0003), but not across site (*p* = 0.87). Pairwise comparisons of richness across depth revealed that *M. pyrifera* communities are significantly richer on blades from bottom depths (*p* < 0.001), while richness is similar on middle and top blades (*p* = 0.64; **Figure 1D**).

We visualized similarity in bacterial community composition with a principle coordinate plot generated from a weighted UniFrac distance matrix, which clearly shows that water column communities are distinct from those on *Macrocystis* (**Figure 1E**). We then tested for differences in community composition between sample types and across *Macrocystis* blades of differing depth with multifactor PERMANOVA that included fixed terms for sample type (*Macrocystis* versus water) and depth (top, middle, and bottom), and site as a random effect (*N* = 55 total). In this full model sample type was highly significant (Pseudo-*F* = 3.33, df = 1, *p* = 0.0001), but depth and site were not (depth: Pseudo-*F* = 2.59, df = 2, *p* = 0.11; site: Pseudo-*F* = 0.73, df = 2, *p* = 0.7) and dispersion differed across all three factors (PERMDISP *p* < 0.05). The substantial differences in community composition between water and *Macrocystis* are apparent in the OTU heatmap and taxa summaries (**Figure 1**). In order to assess differences in community structure within *Macrocystis* epibiota, we conducted a separate PERMANOVA on only *Macrocystis* samples (*N* = 44), again with depth as a fixed factor and site random. Here, depth is a significant determinant of community structure (Pseudo-*F* = 4.31, df = 2, *p* = 0.015), but site is not (Pseudo-*F* = 1.63, df = 2, *p* = 0.09). Pairwise comparisons across depth again show that the epibiota on bottom blades are distinct (p = 0.001), but epibiota on top and middle blades do not differ (*p* = 0.48). Differences across depth are driven by changes in the relative abundance of several phyla — Verrucomicrobia and Alphaproteobacteria are more abundant on upper and middle blades, while Gammaproteobacteria are more abundant on deeper blades (**Figure 1B** and **Supplementary Figure S2**) — and particularly by the turnover of dominant genera within major clades (**Figure 1C**). For instance, *Persicirhabdus* (Verrucomicrobia) is highly enriched in the upper depths, while a shift from Saprospiraceae to *Dokdonia* occurs within Bacteroidetes from deep to shallow. Overall, 21 genera are differentially abundant between bottom and upper depths (FDR adjusted *p* < 0.1; **Supplementary Table S2**). Genera enriched on blades from the bottom depth predominantly fall in the Gammaproteobacteria, including *Psychromonas*, a genus with demonstrated capacity for alginate utilization (**Table 1**). Blades from upper depths were enriched for Sphingomonadales (Alphaproteobacteria), *Dokdonia*, and *Persicirhabdus* (**Figure 1C** and **Supplementary Table S2**).

**Table 1:** Diverse bacterial genera from isolates cultured in the *Macrocystis pyrifera* forest demonstrate alginate utilization.

Genus	Isolates	Observed activity					OTUs^∗^	Reported alginate utilization
		Growth (obligate)	Growth (supplemented)	Degrade	Both	None		
*Alteromonas*	35	-	9%	34%	11%	46%	16	Sawabe et al., 1995; Iwamoto et al., 2001
*Cellulophaga*	9	-	0%	11%	67%	22%	7	Martin et al., 2015; Zhu et al., 2016
*Cobetia*	9	-	44%	22%	11%	22%	6	Martin et al., 2015; Gong et al., 2016
*Colwellia*	3	-	33%	33%	33%	0%	3	^∧^This study
*Formosa*	1	-	0%	100%	0%	0%	1	Mann et al., 2013; Tanaka et al., 2015
*Glaciecola*	2	-	50%	50%	0%	0%	2	Xu et al., 2017
*Halomonas*	10	-	10%	10%	20%	60%	8	Wong et al., 2000
*Idiomarina*	5	-	20%	0%	20%	60%	3	^∧^This study
*Litoreibacter*	1	-	0%	0%	0%	100%	1	
*Maribacter*	5	-	0%	80%	0%	20%	5	Nedashkovskaya et al., 2004; Zhan et al., 2017
*Marinobacter*	2	-	0%	0%	0%	100%	1	
*Paraglaciecola*	13	-	8%	38%	31%	23%	9	Schultz-Johansen et al., 2016
*Photobacterium*	2	-	50%	50%	0%	0%	1	Wong et al., 2000
*Polaribacter*	1	-	100%	0%	0%	0%	1	Dong et al., 2012; Tanaka et al., 2015
*Pseudoalteromonas*	94	-	14%	46%	18%	22%	44	Matsushima et al., 2010; Li et al., 2011
*Pseudomonas*	4	-	0%	0%	0%	100%	2	Boyd et al., 1993
*Psychrobacter*	2	-	50%	0%	0%	50%	2	Dong et al., 2012
*Psychromonas*	3	-	33%	33%	33%	0%	1	Dong et al., 2012
*Rheinheimera*	2	-	0%	0%	100%	0%	1	^∧^This study
*Rhodococcus*	1	-	0%	0%	0%	100%	1	
*Shewanella*	2	-	50%	50%	0%	0%	2	Martin et al., 2015; Tanaka et al., 2015
*Sulfitobacter*	26	-	8%	19%	4%	69%	8	Ivanova et al., 2004
*Tamlana*	1	+	0%	0%	100%	0%	1	Tanaka et al., 2015
*Vibrio*	22	+	5%	50%	18%	27%	12	Han et al., 2004; Martin et al., 2015
Total	255		13%	36%	17%	34%	138	

*^∗^OTUs represent isolates within each genus clustered at 99.8–100% similarity in 16S rRNA sequences. ^∧^We identified three bacterial genera that were not previously known to use alginate (Colwellia, Idiomarina, and Rheinheimera).*

### Carbohydrate Metabolic Profiles

**FIGURE 2:**
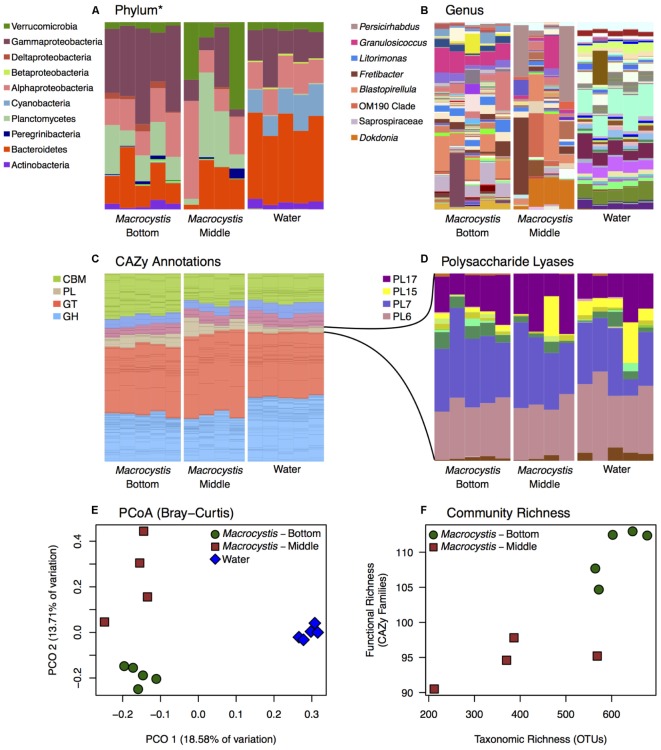
Bacterial taxonomic and functional profiles within a single kelp bed. Bacterial communities from kelp blades at middle (*n* = 4) and bottom (*n* = 5) depths, and in the surrounding water column (*n* = 5), in one kelp bed (Site 3). **(A,B)** Taxonomic profiles from 16S rRNA gene amplicon sequencing are variable between water and *Macrocystis*, and across depth. See **Supplementary Figure S6** for corresponding taxonomic profiles derived from metagenomic data. **(A)** Phylum (^∗^Class for Proteobacteria) level. **(B)** Genus level (most abundant genera shown in legend). **(C,D)** Relative abundance of Carbohydrate Active Enzyme (CAZy) annotations from these communities is largely consistent across sample types. **(C)** CAZy annotations largely fall within Carbohydrate Binding Modules (CBM), Glycosyl Transferases (GT), Glycoside Hydrolases (GH), and Polysaccharide Lyase classes (PL). Enzyme families within these broader groups are represented by stacked bars of the same color. **(D)** Relative abundance of CAZy families within the Polysaccharide Lyase class. Families PL6, 7, 15, and 17 are the most abundant and almost exclusively contain alginate and oligoalginate lyases. **(E)** Principle coordinate plot of CAZy annotations constructed from Bray–Curtis dissimilarity matrix shows differences between *Macrocystis* bottom and middle blades, and the water column, similar to taxonomic composition (statistical tests summarized in **Table 2**). **(F)** Functional richness (number of CAZy families) is correlated with taxonomic richness of OTUs in *Macrocystis* communities (*r*_s_ = 0.87).

We reconstructed carbohydrate metabolic profiles for *M. pyrifera* and water column samples from one kelp bed (Site 3), using shotgun metagenomic sequencing, to assess the capacity for alginate utilization in these bacterial communities (**Figure 2**). Metagenomic reads were annotated using the Carbohydrate Active Enzymes database (CAZy; release 2014.09.04; Lombard et al., 2014). The majority of CAZy annotations detected on *M. pyrifera* and in water column bacterial communities fall within Glycoside Hydrolase and Glycosyl Transferase enzymes (**Figure 2C**). Overall, metagenomic reads mapping to CAZy genes were assigned to enzyme families associated with the metabolism of diverse algal carbohydrates, in addition to the metabolism of substrates related to fundamental bacterial processes (e.g., peptidoglycan, lipopolysaccharide; CAZypedia Consortium, 2017; **Supplementary Table S3**).

Most CAZy families are found in similar proportions across kelp and water column bacterial communities (**Figure 2C**), despite the substantial taxonomic differences observed (**Figures 1**, **2A,B**). The Polysaccharide Lyase (PL) enzyme class, which includes alginate and oligoalginate lyases, represents a small portion of the carbohydrate functional profiles, 5–10% overall (**Figure 2C**). However, the majority of PL genes belong to the PL6, 7, 15, and 17 families (**Figure 2D**) within both kelp and water column communities. The only known activities in these families are alginate and oligoalginate lyases, with the exception of PL6 which also contains chondroitinase B. Notably, three of these families (PL6, 7, 17) are among the most abundant CAZy families in the *Macrocystis* epibiota and together represent ∼5% of the total carbohydrate metabolic profile (**Figure 2** and **Supplementary Table S3**). The same polysaccharide lyase families are found in the water column, though they make up less of the relative abundance of the overall CAZy profile (**Figure 2**). Indeed, we found 4 CAZy families to be differentially enriched between *Macrocystis* communities and the water column (**Supplementary Table S2**), with all four families enriched on the *Macrocystis* surface. Notably, two of these families contain alginate lyase enzymes (PL7 and PL17), while a third (CBM32) has been shown to be involved in the binding of alginate by polysaccharide lyases (Sim et al., 2017; Lyu et al., 2018).

Functional profiles of CAZy genes differ in composition between *M. pyrifera* and the water column, and across blade depths, consistent with the observed patterns of taxonomic composition. We compared functional composition across sample types with PERMANOVA analysis of Bray–Curtis distance matrices and visualized differences with PCoA plots (**Figure 2E**). The profiles of carbohydrate metabolic potential differ significantly between the water column and *Macrocystis* epibiota (PERMANOVA: Pseudo-*F* = 1.92, df = 1, *p* = 0.0006) and the *Macrocystis* communities are significantly more dispersed (PERMDISP: *p* = 0.0005) (**Table 2**). Comparing *Macrocystis* samples between middle and bottom depth reveals significant differences in functional composition (PERMANOVA: Pseudo-*F* = 1.3, df = 1, *p* = 0.0066), while dispersion does not differ (**Table 2**). In contrast, we see no difference in functional richness between *Macrocystis* and water column communities at the gene (Welch’s *t*-test for observed genes: *p* = 0.18) or family level (Welch’s *t*-test for observed families: *p* = 0.33; **Table 2**). *Macrocystis*-associated communities from bottom depths do harbor greater CAZy richness at the family level (Welch’s *t*-test *p* = 0.0023; **Table 2**), while depths do not differ in CAZy gene richness (Welch’s *t*-test *p* = 0.23; **Table 2**). *Macrocystis* blade communities at the bottom depth were also taxonomically richer, and we find that *Macrocystis*-associated community functional richness (CAZy families) is positively correlated with taxonomic richness (Spearman’s rank correlation test: *p* = 0.0045, *r*_s_ = 0.87; **Figure 2F**).

**Table 2:** Summary of statistical tests comparing bacterial carbohydrate metabolic profiles.

Data	Comparison		Welch’s *t*-test Observed genes			Welch’s *t*-test Observed families		
	Group 1	Group 2	df	*t*	*p*	df	*t*	*p*
**(A) CAZy functional profile richness**								
All samples	Blade (*n* = 9)	Water (*n* = 5)	11.61	-1.43	0.18	4.78	1.07	0.33
*Macrocystis*	Bottom (*n* = 5)	Middle (*n* = 4)	9.86	-1.27	0.23	6.98	6.95	0.0023
**(B) CAZy functional profile dissimilarity**								
All samples	Blade (*n* = 9)	Water (*n* = 5)	1	1.92	0.0006	1,12	67.65	0.0005
*Macrocystis*	Bottom (*n* = 5)	Middle (*n* = 4)	1	1.3	0.0066	1,7	4.78	0.087

We hypothesized that alginate metabolic capacity might differ with depth in the *Macrocystis* epibiota, given the significant difference in functional community structure across depth and the prevalence of several alginate lyase families within carbohydrate metabolic profiles. However, no genes encoding alginate degrading enzymes or PL families are differentially enriched across blade depths. We next looked at the total metabolic profiles to identify metabolic processes that might be associated with the observed depth patterning. While no CAZy genes were differentially enriched, we identified three CAZy families (GH16, CBM6, and CBM13) that were enriched in the bottom depth (adjusted *p* < 0.1; **Supplementary Table S2**). These families contain enzymes involved in the hydrolysis of diverse algal polymers (GH16), in particular cellulose (CBM6, CBM13; Adams et al., 2011).

### Alginate Utilizing Bacteria

**FIGURE 3:**
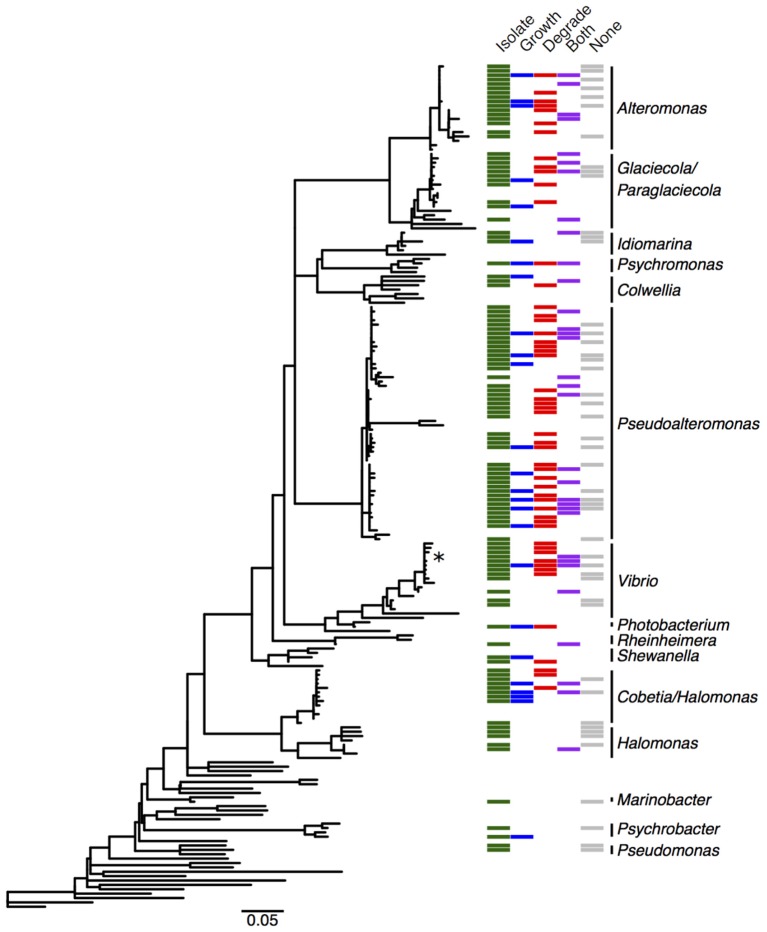
Distribution of alginate utilizing taxa within the Gammaproteobacteria. Phylogenetic tree of Gammaproteobacteria genera detected in the *M. pyrifera* epibiota and surrounding water column in this study, constructed from 16S rRNA gene sequences by placing cultured bacterial isolates into a tree of reference sequences with RAxML EPA. See **Supplementary Figure S3** for labeled tree with isolate identities, reference taxa, and accession numbers. Green: cultured bacterial isolates in this study; sequences from isolated bacteria with 99.8–100% 16S similarity were collapsed into operational taxonomic units (OTUs). Blue: cultured isolates from this study whose growth was enriched by at least 50% in the presence of alginate, or that grew with alginate as the sole carbon source (marked with ^∗^). Red: cultured isolates in this study that degrade alginate. Purple: cultured isolates whose growth was enhanced and that degrade alginate. Gray: cultured isolates demonstrating neither enhanced growth nor degradation. Detailed information on cultured isolates in **Supplementary Table S1**.

We cultured bacteria from a subset of the kelp blades and water samples using tryptone and alginate marine media. A total of 338 isolates were assayed for the utilization of alginate, and 255 of these were successfully identified by Sanger sequencing the V4–V9 region of the 16S rRNA. Of these 255 isolates (**Table 1**), 91 were capable of depolymerizing alginate (36%), 33 were capable of metabolizing it for growth (13%), and 44 could do both (17%). A larger proportion of bacteria isolated on alginate media (76%) demonstrated alginate utilization compared to bacteria isolated on tryptone media (55%). Bacterial isolates were predominantly from the Gammaproteobacteria (**Figure 3** and **Table 1**), with *Pseudoalteromonas* being the most abundant cultured genus (94/255 isolates) and containing the most active isolates (*n* = 73), followed by *Alteromonas* and *Vibrio*. Two isolates were capable of obligate growth on alginate; these were assigned to *Tamlana* (Flavobacteriaceae) and *Vibrio* (Gammaproteobacteria). Finally, while alginate utilization has been reported for most assayed genera previously (**Table 1**), we observed this capacity for the first time in three genera: *Colwellia, Idiomarina*, and *Rheinheimera*.

To further understand how alginate utilization varies across closely related taxa, Sanger sequences from the 255 isolates were clustered at 100% similarity into OTUs. The resulting 138 OTUs fall in 24 genera within the Gammaproteobacteria, Alphaproteobacteria, and Flavobacteriaceae (**Table 1**). OTU representative sequences were aligned to the SILVA 128 reference database and placed into phylogenetic trees for each of these clades (**Figure 3** and **Supplementary Figures S3**–**S5**). In total, 104 OTUs (representing 20 genera) demonstrated alginate utilization (**Supplementary Table S1**). Importantly, 17 OTUs were composed of isolates with and without this capacity; these taxa were thus discordant for alginate utilization despite identical 16S sequences. Discordance was also observed for type of activity (metabolism, degradation, both; **Supplementary Table S1**).

To deeply survey the total cultured bacteria from *M. pyrifera* and the water column, and to investigate how representative the isolated taxa were, we sequenced bacterial colonies from 24 culture plates (18 kelp; 6 water). Plates were scraped for all colonies and sequenced at the V4 region of the 16S rRNA gene on the Illumina MiSeq platform. Sequences were clustered into 150 OTUs that fall within 62 genera. Cultured taxa belong almost entirely to the Gammaproteobacteria, Alphaproteobacteria, and Flavobacteriaceae (**Figure 1A** and **Supplementary Figure S1**). Planctomycetes and Verrucomicrobia, which were abundant in the culture-independent dataset, were absent among cultured taxa. Notably, the three genera (*Alteromonas, Pseudoalteromonas*, and *Vibrio*) comprising the majority of cultured taxa occur at very low abundance in the *M. pyrifera* epibiota (**Supplementary Figure S1**). Overall, there is little overlap between the cultured and uncultured bacterial communities (**Figure 1A**).

## Discussion

### Structure and Diversity of the *Macrocystis pyrifera* Epibiota

The epibiota of *M. pyrifera* (**Figure 1** and **Supplementary Figure S2**) is composed of bacteria known to be prevalent in the marine environment (Wahl et al., 2012; Singh and Reddy, 2014) and on algal surfaces (Staufenberger et al., 2008; Burke et al., 2011; Michelou et al., 2013). We find distinct bacterial communities on *Macrocystis* compared to the water column, though large numbers of OTUs are shared across both, consistent with other kelps (Lemay et al., 2018). The Planctomycetes phylum in particular was highly enriched relative to the water column (**Figure 1B**), and is commonly found on kelp (Vollmers et al., 2017; Lemay et al., 2018). Planctomycetes are often underrepresented in amplicon sequencing studies, but were found to dominate biofilms on *Laminaria* (Bengtsson and Øvreås, 2010). *Persicirhabdus* and *Rubritalea* (Verrucomicrobia) are also emerging as common associates of *Macrocystis* (**Figure 1C**) and kelp more generally (Vollmers et al., 2017).

We observed that epibiotic communities differ in composition and richness across blade depths (**Figure 1** and **Supplementary Figure S2**), with richer communities being found at the bottom depth (**Figure 1D**). This depth associated structuring of bacterial communities represents a novel axis of community variation in *Macrocystis* forests. Blades at lower depths are older (Wheeler and North, 1981; Reed et al., 2015), and while this age difference may only correspond to several months (Druehl and Wheeler, 1986; Van Tussenbroek, 1989), this pattern is consistent with work in other kelp documenting microbial succession and increasing richness as blades age (Bengtsson et al., 2012), as well as microbiota changes with tissue degradation and disease state (Marzinelli et al., 2015). Additionally, we did not observe such differences between water column communities across depths, which would indicate that abiotic factors are not driving community variation. However, due to an unbalanced distribution of water samples across sites, these observations were not statistically validated. Regardless, the increasing diversity of bacteria and functional genes involved in carbohydrate metabolism observed on deeper blades is likely a consequence of reduced production of anti-fouling metabolites, and other processes during senescence, that make the host surface increasingly permissive to colonization and degradation (Egan et al., 2013).

The taxa that are enriched at lower depths (**Figure 1** and **Supplementary Table S2**) may be opportunistic metabolisers of kelp tissue. Interestingly, several Gammaproteobacteria genera were enriched in deeper communities and this may be explained by their capacity for degrading diverse algal polysaccharides (Michel et al., 2006), making them well suited as generalists on older tissue. Further, the substantial enrichment of Saprospiraceae on lower depth blades may reflect a similar role for this group in tissue degradation, as this family includes known degraders of algal polysaccharides (McIlroy and Nielsen, 2014). Younger tissue may be more likely to host taxa specifically associated with kelps, and we find several abundant taxa that are detected on kelps in other studies, such as *Persicirhabdus* (Vollmers et al., 2017; Chen and Parfrey, 2018) and *Dokdonia* (**Figure 1C**), which is often found on seaweeds generally. Taken together, these patterns are consistent with succession on *M. pyrifera* blades with increasing depth. However, as depth is additionally associated with light reduction and variations in *Macrocystis* tissue physiology (Konotchick et al., 2013), the exact cause of this patterning remains unclear. Furthermore, the tidal range at these kelp beds was high during the period of collection (>3 m) and blades at the middle depth were likely at the surface at some points during the day. Thus the exposure to similar abiotic factors, such as air temperature, sunlight, and oxygen, maybe have also contributed to the similarity observed between shallower communities relative to those at depth.

### Epibiotic Functional Profiles Change With Depth

Carbohydrate metabolism in the *Macrocystis* forest appears to be structured around the major constituents of kelp tissue. Of the most abundant CAZy families that we detected, many represent activities related to carbohydrate substrates in *Macrocystis* (**Supplementary Table S3**; Ravanal et al., 2016), in particular alginate and cellulose. Despite their taxonomic differences, kelp and water column metabolic profiles appear compositionally similar at the level of CAZy families (**Figure 2C** and **Supplementary Table S3**). Yet, we did observe differences between carbohydrate metabolic profiles with regard to alginate metabolism. Interestingly, two CAZy families containing alginate lyase enzymes, and one family shown to be involved in binding alginate, were enriched on the *Macrocystis* surface relative to the water column (**Supplementary Table S2**). This may reflect an abundance of alginate on the kelp surface relative to the water column and could underlie some of the taxonomic differences observed between kelp and water bacterial communities. However, the pre-filtering of water column samples prior to processing (thereby removing larger detrital material), in addition to the highly disparate sequencing coverage of the metagenomic samples and unbalanced distribution of water samples across depths, necessitate further validation of these observations.

The capacity for alginate utilization was observed across all bacterial communities (**Figure 2D**) and the majority of all PL genes fall within four families that almost exclusively contain alginate and oligoalginate lyases (Jagtap et al., 2014). Interestingly, these same families were shown by Hehemann et al. (2016) to contain sufficient metabolic diversity to partition alginate within bacterial populations. Furthermore, the abundance of three of these families (PL7, 6, 17) in epibiotic functional profiles indicates the importance of alginate as a carbon source in this system. Finally, the presence of these families across taxonomically diverse bacterial communities (**Figures 2A,B**) indicates a broad capacity for alginate utilization, particularly as the cultured taxa we assayed are underrepresented in these communities. While inferences drawn by comparing across these different datasets are understandably limited, the correspondence observed between taxa summaries generated from both metagenomic and amplicon sequences (**Supplementary Figure S6**) supports the observation of taxonomic variability across these samples. Interestingly, many of the genes within these PL families are annotated to taxa that were also cultured and demonstrated the capacity for alginate utilization, though conversely, alginolytic genes assigned to numerous uncultured genera were also present in the functional profiles. The annotation of alginate lyase genes from diverse taxa may also indicate the potential for broad distribution of this capacity, however, these results alone do not conclusively demonstrate the presence of alginate utilization in genera other than those that were assayed. While bacteria that utilize alginate may persist on the algal surface at lower abundances or as opportunists, further work is required to conclusively determine how prevalent this metabolic capacity is.

We report a depth associated structuring of epibiotic functional profiles consistent with the observed taxonomic structuring. Communities on deeper blades are compositionally different (**Figures 2C–E**) and host functionally and taxonomically richer assemblages (**Figure 2F**). These observations are consistent with dynamics of bacterial succession, whereby poorly defended tissue is increasingly colonized by generalist taxa. Despite these differences, no CAZy genes or families related to alginate metabolism were differentially abundant across blade depths. Given the magnitude of alginate in this system, this may indicate the occurrence of constitutive, rather than variable alginate turnover. Possibly, only a subset of bacteria on the *Macrocystis* surface metabolize alginate, regardless of tissue state and community composition, and this activity is sufficient to liberate alginate carbon. It should be noted that the lack of differentially abundant CAZy genes observed attests only to the gene content of these communities and does not reflect variation in gene expression that may be present across depths. Thus the capacity for alginate utilization may be present within all functional profiles, while a depth associated patterning of activity may only be apparent from gene expression data. While it remains unclear whether alginate metabolism underlies the differences between epibiotic communities, it may be integral to structuring assemblages on detrital material, particularly as detritus accounts for the majority of kelp carbon export (Krumhansl and Scheibling, 2012). As stated previously, the filtering of water samples in this work precludes a robust characterization of detrital-associated assemblages. Future work should aim to determine whether these bacterial communities are in fact structured around the utilization of kelp derived alginate.

We did detect the enrichment of three CAZy families (GH16, CBM6, CBM13; **Supplementary Table S2**) in communities on deeper blades and these contain enzymes targeting several algal carbohydrates, particularly cellulose (Adams et al., 2011). As cellulose is the second most abundant carbohydrate in *Macrocystis* (Ravanal et al., 2016), this may reflect a more complete degradation of algal tissue occurring on deeper blades, supporting a successional role for these communities. Interestingly, alginate degradation prior to cellulose saccharification improves carbohydrate liberation in *Macrocystis* tissue (Ravanal et al., 2016). Constitutive alginate turnover may therefore be advantageous, as it would increase access to other carbon substrates once host defenses are reduced. In this case, successional bacterial communities would be structured around the availability of other carbon substrates, as opposed to alginate.

### Diverse Cultured Bacteria in *Macrocystis* Forests Are Capable of Utilizing Alginate

A large proportion of isolates cultured from the epibiota of *M. pyrifera* and surrounding water column are capable of degrading and/or metabolizing alginate (66%; **Table 1**). The majority of isolates cultured on both selective (containing alginate) and non-selective media demonstrated this activity, indicating that this pattern is not simply the product of media bias. The capacity for alginate utilization is broadly, but unevenly, distributed across the major taxonomic groups cultured here (Gammaproteobacteria: **Figure 3** and **Supplementary Figure S3**, Alphaproteobacteria: **Supplementary Figure S4**, and Flavobacteriaceae: **Supplementary Figure S5**), all of which are important marine carbon metabolisers (Edwards et al., 2010; Teeling et al., 2016). Most of the taxa capable of utilizing alginate have been reported in previous studies, however, we extend this catalog of genera by three, all within the Gammaproteobacteria (**Table 1**).

The capacity for alginate utilization, and polysaccharide degradation more generally, is likely more widespread than estimated by culturing alone (Bengtsson et al., 2011; Martin et al., 2015). The poor representation of cultured genera in *M. pyrifera* epibiotic communities (**Figure 1A**) is consistent with previous studies, and this culturing inefficiency evidently biases characterizing metabolic activity, such as alginate utilization, toward underestimation. Indeed, the vast majority of taxa detected by sequencing uncultured communities were not isolated and assayed, though genes involved in alginate metabolism (PL families 6, 7, 15, 17 in **Figure 2D**) are consistently present and reasonably abundant across all communities. Specifically, the Planctomycetes and Verrucomicrobia are well represented in epibiotic communities but are entirely absent among cultured taxa (**Supplementary Figure S1**). These groups may participate in important interactions with the algal host (Lage and Bondoso, 2011; Vollmers et al., 2017), including carbon metabolism (Bengtsson and Øvreås, 2010), yet are historically difficult to culture (Op den Camp et al., 2009; Fuerst and Sagulenko, 2011).

The patchy phylogenetic distribution of observed alginate utilization may result from horizontal gene transfer (HGT), which would further support the potential for diverse taxa to possess this capacity. Marine bacteria are capable of highly frequent HGT (McDaniel et al., 2010) and the transfer of alginate lyase genes has been reported previously (Wargacki et al., 2012; Hehemann et al., 2016; Zhu et al., 2017). Simple carbohydrate metabolism is also often shallowly conserved within bacterial phylogenies (Martiny et al., 2013), likely owing to the relative ease of transferring genes required for low complexity metabolic pathways. The abundance of alginate in kelp forests coupled with its relatively simple metabolic requirements could therefore produce a broad incorporation of alginate lyase genes. Interestingly, many of the genera that demonstrate alginate utilization in this study also metabolize numerous other algal polysaccharides (Michel et al., 2006; Alderkamp et al., 2007; Mann et al., 2013; Schultz-Johansen et al., 2016). This metabolic heterogeneity and the diversity of observed alginate utilizing taxa both support a disconnect between 16S identity and simple carbohydrate metabolism that is consistent with HGT.

We demonstrate here that the *M. pyrifera* epibiota and surrounding water column harbor diverse culturable bacteria capable of degrading and metabolizing alginate, a function that is integral to the cycling of kelp carbon in the coastal ocean. The capacity for alginate utilization appears to have a broad, but patchy distribution in bacterial phylogenies; it is not always present within a given genus and is variable even across isolates with identical 16S rRNA sequences. This indicates a weak association between functional capacity and taxonomy at high resolution. While the prevalence and distribution of alginate utilization across taxa within the *M. pyrifera* epibiota remains unclear, the pervasiveness of this trait within cultured bacteria, abundance of alginate metabolic genes assigned to diverse genera, and presence of these genes across taxonomically variable bacterial communities, suggests that it may be common and the cycling of algal carbon is performed by diverse groups of marine bacteria.

## Author Contributions

ML, LP, and JL designed the project. JL conducted the assays of cultured samples, generated the sequencing data for cultured and uncultured samples, and analyzed the data with input from ML and LP. JL wrote the first draft of the manuscript. All authors contributed to the revisions.

## Conflict of Interest Statement

The authors declare that the research was conducted in the absence of any commercial or financial relationships that could be construed as a potential conflict of interest.

## Footnotes

1 http://www.earthmicrobiome.org/protocols-and-standards/16s/

2 http://hannonlab.cshl.edu/fastx_toolkit/

3 www.ebi.ac.uk

4 www.ncbi.nlm.nih.gov/genbank/

## References

[B1] Adams A. S. Jordan M. S. Adams S. M. Suen G. Goodwin L. A. Davenport K. W. (2011). Cellulose-degrading bacteria associated with the invasive woodwasp *Sirex noctilio*. *ISME J.* 5 1323–1331. 10.1038/ismej.2011.14 21368904 PMC3146269

[B2] Alderkamp A. C. Van Rijssel M. Bolhuis H. (2007). Characterization of marine bacteria and the activity of their enzyme systems involved in degradation of the algal storage glucan laminarin. *FEMS Microbiol. Ecol.* 59 108–117. 10.1111/j.1574-6941.2006.00219.x 17233748

[B3] Anderson M. J. (2004). *PERMDISP: a FORTRAN Computer Program for Permutational Analysis of Multivariate Dispersions (for Any Two-Factor ANOVA Design) Using Permutation Tests.* Auckland: Department of Statistics, University of Auckland.

[B4] Anderson M. J. Gorley R. N. Clarke R. K. (2005). *Permanova. Permutational Multivariate Analysis of Variance, a Computer Program.* Auckland: Department of Statistics, University of Auckland, 24.

[B5] Azam F. Malfatti F. (2007). Microbial structuring of marine ecosystems. *Nat. Rev. Microbiol.* 5 782–791. 10.1038/nrmicro1747 17853906

[B6] Bengtsson M. M. Øvreås L. (2010). Planctomycetes dominate biofilms on surfaces of the kelp *Laminaria hyperborea*. *BMC Microbiol.* 10:261. 10.1186/1471-2180-10-261 20950420 PMC2964680

[B7] Bengtsson M. M. Sjøtun K. Lanzén A. Øvreås L. (2012). Bacterial diversity in relation to secondary production and succession on surfaces of the kelp *Laminaria hyperborea*. *ISME J.* 6 2188–2198. 10.1038/ismej.2012.67 22763650 PMC3505018

[B8] Bengtsson M. M. Sjøtun K. Storesund J. E. Øvreås L. (2011). Utilization of kelp-derived carbon sources by kelp surface-associated bacteria. *Aquat. Microb. Ecol.* 62 191–199. 10.3354/ame01477

[B9] Benjamini Y. Hochberg Y. (1995). Controlling the false discovery rate: a practical and powerful approach to multiple testing. *J. R. Stat. Soc. B* 57 289–300.

[B10] Berger S. A. Krompass D. Stamatakis A. (2011). Performance, accuracy, and web server for evolutionary placement of short sequence reads under maximum likelihood. *Syst. Biol.* 60 291–302. 10.1093/sysbio/syr010 21436105 PMC3078422

[B11] Boyd A. Ghosh M. May T. B. Shinabarger D. Keogh R. Chakrabarty A. M. (1993). Sequence of the algL gene of *Pseudomonas aeruginosa* and purification of its alginate lyase product. *Gene* 131 1–8. 10.1016/0378-1119(93)90662-M 8370530

[B12] Bray J. R. Curtis J. T. (1957). An ordination of the upland forest communities of southern Wisconsin. *Ecol. Monogr.* 27 325–349. 10.2307/1942268

[B13] Burke C. Thomas T. Lewis M. Steinberg P. Kjelleberg S. (2011). Composition, uniqueness and variability of the epiphytic bacterial community of the green alga Ulva australis. *ISME J.* 5 590–600. 10.1038/ismej.2010.164 21048801 PMC3105733

[B14] Camacho C. Coulouris G. Avagyan V. Ma N. Papadopoulos J. Bealer K. (2009). BLAST+: architecture and applications. *BMC Bioinformatics* 10:421. 10.1186/1471-2105-10-421 20003500 PMC2803857

[B15] Caporaso J. G. Bittinger K. Bushman F. D. DeSantis T. Z. Andersen G. L. Knight R. (2010a). PyNAST: a flexible tool for aligning sequences to a template alignment. *Bioinformatics* 26 266–267. 10.1093/bioinformatics/btp636 19914921 PMC2804299

[B16] Caporaso J. G. Kuczynski J. Stombaugh J. Bittinger K. Bushman F. D. Costello E. K. (2010b). QIIME allows analysis of high-throughput community sequencing data. *Nat. Methods* 7 335–336. 10.1038/nmeth.f.303 20383131 PMC3156573

[B17] Caporaso J. G. Lauber C. L. Walters W. A. Berg-Lyons D. Huntley J. Fierer N. (2012). Ultra-high-throughput microbial community analysis on the Illumina HiSeq and MiSeq platforms. *ISME J.* 6 1621–1624. 10.1038/ismej.2012.8 22402401 PMC3400413

[B18] CAZypedia Consortium (2017). Ten years of CAZypedia: a living encyclopedia of carbohydrate-active enzymes. *Glycobiology* 28 3–8. 29040563 10.1093/glycob/cwx089

[B19] Chao A. (1984). Nonparametric estimation of the number of classes in a population. *Scand. J. Stat.* 11 265–270.

[B20] Chen M. Y. Parfrey L. W. (2018). Incubation with macroalgae induces large shifts in water column microbiota, but minor changes to the epibiota of co-occurring macroalgae. *Mol. Ecol.* 27 1966–1979. 10.1111/mec.14548 29524281

[B21] Clarke K. R. Gorley R. N. (2006). *PRIMER v6: User Manual/Tutorial.* Plymouth: PRIMER-E.

[B22] Clasen J. L. Shurin J. B. (2017). Kelp forest size alters microbial community structure and function on Vancouver Island, Canada. *Ecology* 96 862–872. 10.1890/13-2147.1 26236881

[B23] Dong S. Yang J. Zhang X. Y. Shi M. Song X. Y. Chen X. L. (2012). Cultivable alginate lyase-excreting bacteria associated with the arctic brown alga Laminaria. *Mar. Drugs* 10 2481–2491. 10.3390/md10112481 23203272 PMC3509530

[B24] Druehl L. D. Wheeler W. N. (1986). Population biology of *Macrocystis integrifolia* from British Columbia, Canada. *Mar. Biol.* 90 173–179. 10.1007/BF00569124

[B25] Edgar R. C. (2004). MUSCLE: multiple sequence alignment with high accuracy and high throughput. *Nucleic Acids Res.* 32 1792–1797. 10.1093/nar/gkh340 15034147 PMC390337

[B26] Edgar R. C. (2010). Search and clustering orders of magnitude faster than BLAST. *Bioinformatics* 26 2460–2461. 10.1093/bioinformatics/btq461 20709691

[B27] Edwards J. L. Smith D. L. Connolly J. McDonald J. E. Cox M. J. Joint I. (2010). Identification of carbohydrate metabolism genes in the metagenome of a marine biofilm community shown to be dominated by Gammaproteobacteria and Bacteroidetes. *Genes* 1 371–384. 10.3390/genes1030371 24710093 PMC3966224

[B28] Egan S. Harder T. Burke C. Steinberg P. Kjelleberg S. Thomas T. (2013). The seaweed holobiont: understanding seaweed–bacteria interactions. *FEMS Microbiol. Rev.* 37 462–476. 10.1111/1574-6976.12011 23157386

[B29] Eren A. M. Maignien L. Sul W. J. Murphy L. G. Grim S. L. Morrison H. G. (2013). Oligotyping: differentiating between closely related microbial taxa using 16S rRNA gene data. *Methods Ecol. Evol.* 4 1111–1119. 10.1111/2041-210X.12114 24358444 PMC3864673

[B30] Eren A. M. Morrison H. G. Lescault P. J. Reveillaud J. Vineis J. H. Sogin M. L. (2017). Minimum entropy decomposition: unsupervised oligotyping for sensitive partitioning of high-throughput marker gene sequences. *ISME J.* 9 968–979. 10.1038/ismej.2014.195 25325381 PMC4817710

[B31] Fuerst J. A. Sagulenko E. (2011). Beyond the bacterium: planctomycetes challenge our concepts of microbial structure and function. *Nat. Rev. Microbiol.* 9 403–413. 10.1038/nrmicro2578 21572457

[B32] Gong J. S. Liu X. M. Zhang M. J. Li H. Geng Y. Li H. (2016). Purification and characterization of a high salt-tolerant alginate lyase from *Cobetia* sp. WG-007. *Biotechnol. Appl. Biochem.* 64 519–524. 10.1002/bab.1506 27189415

[B33] Han F. Gong Q. H. Song K. Li J. B. Yu W. G. (2004). Cloning, sequence analysis and expression of gene alyVI encoding alginate lyase from marine bacterium *Vibrio* sp. QY101. *DNA Seq.* 15 344–350. 10.1080/10425170400019300 15621659

[B34] Han W. Gu J. Cheng Y. Liu H. Li Y. Li F. (2016). Novel alginate lyase (Aly5) from a polysaccharide-degrading marine bacterium, Flammeovirga strain MY04: effects of module truncation on biochemical characteristics, alginate degradation patterns, and oligosaccharide-yielding properties. *Appl. Environ. Microbiol.* 82 364–374. 10.1128/AEM.03022-15 26519393 PMC4702622

[B35] Hehemann J. H. Arevalo P. Datta M. S. Yu X. Corzett C. H. Henschel A. (2016). Adaptive radiation by waves of gene transfer leads to fine-scale resource partitioning in marine microbes. *Nat. Commun.* 7:12860. 10.1038/ncomms12860 27653556 PMC5036157

[B36] Hisano T. Yonemoto Y. Yamashita T. Fukuda Y. Kimura A. Murata K. (1995). Direct uptake of alginate molecules through a pit on the bacterial cell surface: a novel mechanism for the uptake of macromolecules. *J. Ferment. Bioeng.* 79 538–544. 10.1016/0922-338X(95)94744-C

[B37] Ivanova E. P. Gorshkova N. M. Sawabe T. Zhukova N. V. Hayashi K. Kurilenko V. V. (2004). *Sulfitobacter delicatus* sp. nov. and *Sulfitobacter dubius* sp. nov., respectively from a starfish (*Stellaster equestris*) and sea grass (*Zostera marina*). *Int. J. Syst. Evol. Microbiol.* 54 475–480. 10.1099/ijs.0.02654-0 15023963

[B38] Iwamoto Y. Araki R. Iriyama K. I. Oda T. Fukuda H. Hayashida S. (2001). Purification and characterization of bifunctional alginate lyase from *Alteromonas* sp. strain no. 272 and its action on saturated oligomeric substrates. *Biosci. Biotechnol. Biochem.* 65 133–142. 10.1271/bbb.65.133 11272816

[B39] Jagtap S. S. Hehemann J. H. Polz M. F. Lee J. K. Zhao H. (2014). Comparative biochemical characterization of three exolytic oligoalginate lyases from *Vibrio splendidus* reveals complementary substrate scope, temperature, and pH adaptations. *Appl. Environ. Microbiol.* 80 4207–4214. 10.1128/AEM.01285-14 24795372 PMC4068682

[B40] Jiao N. Herndl G. J. Hansell D. A. Benner R. Kattner G. Wilhelm S. W. (2010). Microbial production of recalcitrant dissolved organic matter: long-term carbon storage in the global ocean. *Nat. Rev. Microbiol.* 8 593–599. 10.1038/nrmicro2386 20601964

[B41] Kim H. S. Lee C.-G. Lee E. Y. (2011). Alginate lyase: structure, property, and application. *Biotechnol. Bioproc. Eng.* 16:843 10.1007/s12257-011-0352-8

[B42] Kita A. Miura T. Kawata S. Yamaguchi T. Okamura Y. Aki T. (2016). Bacterial community structure and predicted alginate metabolic pathway in an alginate-degrading bacterial consortium. *J. Biosci. Bioeng.* 121 286–292. 10.1016/j.jbiosc.2015.06.014 26199224

[B43] Konotchick T. Dupont C. L. Valas R. E. Badger J. H. Allen A. E. (2013). Transcriptomic analysis of metabolic function in the giant kelp, *Macrocystis pyrifera*, across depth and season. *New Phytol.* 198 398–407. 10.1111/nph.12160 23488966 PMC3644879

[B44] Konwar K. M. Hanson N. W. Bhatia M. P. Kim D. Wu S. J. Hahn A. S. (2017). MetaPathways v2. 5: quantitative functional, taxonomic and usability improvements. *Bioinformatics* 31 3345–3347. 10.1093/bioinformatics/btv361 26076725 PMC4595896

[B45] Krumhansl K. A. Scheibling R. E. (2012). Production and fate of kelp detritus. *Mar. Ecol. Prog. Ser.* 467 281–302. 10.3354/meps09940 17787876

[B46] Kuznetsova A. Brockhoff P. B. Christensen R. H. B. (2017). *Package ‘lmerTest’. R Package Version, 2.* Available at: https://cran.r-project.org/web/packages/lmerTest/lmerTest.pdf

[B47] Lachnit T. Blümel M. Imhoff J. F. Wahl M. (2009). Specific epibacterial communities on macroalgae: phylogeny matters more than habitat. *Aquat. Biol.* 5 181–186. 10.3354/ab00149

[B48] Lage O. M. Bondoso J. (2011). Planctomycetes diversity associated with macroalgae. *FEMS Microbiol. Ecol.* 78 366–375. 10.1111/j.1574-6941.2011.01168.x 21726244

[B49] Lam C. Harder T. (2007). Marine macroalgae affect abundance and community richness of bacterioplankton in close proximity. *J. Phycol.* 43 874–881. 10.1111/j.1529-8817.2007.00385.x

[B50] Lemay M. A. Martone P. T. Keeling P. J. Burt J. M. Krumhansl K. A. Sanders R. D. (2018). Sympatric kelp species share a large portion of their surface bacterial communities. *Environ. Microbiol.* 20 658–670. 10.1111/1462-2920.13993 29124859

[B51] Li J.-W. Dong S. Song J. Li C.-B. Chen X.-L. Xie B.-B. (2011). Purification and characterization of a bifunctional alginate lyase from *Pseudoalteromonas* sp. SM0524. *Mar. Drugs* 9 109–123. 10.3390/md9010109. 21339950 PMC3039154

[B52] Lombard V. Ramulu H. G. Drula E. Coutinho P. M. Henrissat B. (2014). The carbohydrate-active enzymes database (CAZy) in 2013. *Nucleic Acids Res.* 42 D490–D495. 10.1093/nar/gkt1178 24270786 PMC3965031

[B53] Lozupone C. A. Hamady M. Kelley S. T. Knight R. (2007). Quantitative and qualitative beta diversity measures lead to different insights into factors that structure microbial communities. *Appl. Environ. Microbiol.* 73 1576–1585. 10.1128/aem.01996-06 17220268 PMC1828774

[B54] Lyu Q. Zhang K. Zhu Q. Li Z. Liu Y. Fitzek E. (2018). Structural and biochemical characterization of a multidomain alginate lyase reveals a novel role of CBM32 in CAZymes. *Biochim. Biophys. Acta* 1862 1862–1869. 10.1016/j.bbagen.2018.05.024 29864445

[B55] Mann A. J. Hahnke R. L. Huang S. Werner J. Xing P. Barbeyron T. (2013). The genome of the alga-associated marine flavobacterium *Formosa agariphila* KMM 3901T reveals a broad potential for degradation of algal polysaccharides. *Appl. Environ. Microbiol.* 79 6813–6822. 10.1128/AEM.01937-13 23995932 PMC3811500

[B56] Martin M. Barbeyron T. Martin R. Portetelle D. Michel G. Vandenbol M. (2017). The cultivable surface microbiota of the brown alga *Ascophyllum nodosum* is enriched in macroalgal-polysaccharide-degrading bacteria. *Front. Microbiol.* 6:1487. 10.3389/fmicb.2015.01487 26734000 PMC4690005

[B57] Martiny A. C. Treseder K. Pusch G. (2013). Phylogenetic conservatism of functional traits in microorganisms. *ISME J.* 7 830–838. 10.1038/ismej.2012.160 23235290 PMC3603392

[B58] Marzinelli E. M. Campbell A. H. Zozaya Valdes E. Vergés A. Nielsen S. Wernberg T. (2017). Continental-scale variation in seaweed host-associated bacterial communities is a function of host condition, not geography. *Environ. Microbiol.* 17 4078–4088. 10.1111/1462-2920.12972 26148974

[B59] Matsushima R. Danno H. Uchida M. Ishihara K. Suzuki T. Kaneniwa M. (2010). Analysis of extracellular alginate lyase and its gene from a marine bacterial strain, *Pseudoalteromonas atlantica* AR06. *Appl. Microbiol. Biotechnol.* 86 567–576. 10.1007/s00253-009-2278-z 19844705

[B60] McDaniel L. D. Young E. Delaney J. Ruhnau F. Ritchie K. B. Paul J. H. (2010). High frequency of horizontal gene transfer in the oceans. *Science* 330:50. 10.1126/science.1192243 20929803

[B61] McIlroy S. J. Nielsen P. H. (2014). *The Family Saprospiraceae.* Berlin: Springer, 863–889. 10.1007/978-3-642-38954-2_138

[B62] McKee J. W. A. Kavalieris L. Brasch D. J. Brown M. T. Melton L. D. (1992). Alginate content and composition of *Macrocystis pyrifera* from New Zealand. *J. Appl. Phycol.* 4 357–369. 10.1007/BF02185794

[B63] Meddeb-Mouelhi F. Moisan J. K. Beauregard M. (2014). A comparison of plate assay methods for detecting extracellular cellulase and xylanase activity. *Enzyme Microb. Technol.* 66 16–19. 10.1016/j.enzmictec.2014.07.004 25248694

[B64] Michel G. Nyval-Collen P. Barbeyron T. Czjzek M. Helbert W. (2006). Bioconversion of red seaweed galactans: a focus on bacterial agarases and carrageenases. *Appl. Microbiol. Biotechnol.* 71 23–33. 10.1007/s00253-006-0377-7 16550377

[B65] Michel G. Tonon T. Scornet D. Cock J. M. Kloareg B. (2010). The cell wall polysaccharide metabolism of the brown alga *Ectocarpus siliculosus*. Insights into the evolution of extracellular matrix polysaccharides in Eukaryotes. *New Phytol.* 188 82–97. 10.1111/j.1469-8137.2010.03374.x 20618907

[B66] Michelou V. K. Caporaso J. G. Knight R. Palumbi S. R. (2013). The ecology of microbial communities associated with *Macrocystis pyrifera*. *PLoS One* 8:e67480. 10.1371/journal.pone.0067480 23840715 PMC3686729

[B67] Nedashkovskaya O. I. Kim S. B. Han S. K. Lysenko A. M. Rohde M. Rhee M. S. (2004). *Maribacter* gen. nov., a new member of the family Flavobacteriaceae, isolated from marine habitats, containing the species *Maribacter sedimenticola* sp. nov., *Maribacter aquivivus* sp. nov., *Maribacter orientalis* sp. nov. and *Maribacter ulvicola* sp. nov. *Int. J. Syst. Evol. Microbiol.* 54 1017–1023. 10.1099/ijs.0.02849-0 15280264

[B68] Op den Camp H. J. Islam T. Stott M. B. Harhangi H. R. Hynes A. Schouten S. (2009). Environmental, genomic and taxonomic perspectives on methanotrophic Verrucomicrobia. *Environ. Microbiol. Rep.* 1 293–306. 10.1111/j.1758-2229.2009.00022.x 23765882

[B69] Price M. N. Dehal P. S. Arkin A. P. (2010). FastTree 2–approximately maximum-likelihood trees for large alignments. *PLoS One* 5:e9490. 10.1371/journal.pone.0009490 20224823 PMC2835736

[B70] Pruesse E. Peplies J. Glöckner F. O. (2012). SINA: accurate high-throughput multiple sequence alignment of ribosomal RNA genes. *Bioinformatics* 28 1823–1829. 10.1093/bioinformatics/bts252 22556368 PMC3389763

[B71] Quast C. Pruesse E. Yilmaz P. Gerken J. Schweer T. Yarza P. (2012). The SILVA ribosomal RNA gene database project: improved data processing and web-based tools. *Nucleic Acids Res.* 41 D590–D596. 10.1093/nar/gks1219 23193283 PMC3531112

[B72] R Core Team (2017). *R: A Language and Environment for Statistical Computing.* Vienna: R Foundation for Statistical Computing.

[B73] Ravanal M. C. Pezoa-Conte R. von Schoultz S. Hemming J. Salazar O. Anugwom I. (2016). Comparison of different types of pretreatment and enzymatic saccharification of *Macrocystis pyrifera* for the production of biofuel. *Algal Res.* 13 141–147. 10.1016/j.algal.2015.11.023

[B74] Ravanal M. C. Sharma S. Gimpel J. Reveco-Urzua F. E. Øverland M. Horn S. J. (2017). The role of alginate lyases in the enzymatic saccharification of brown macroalgae, *Macrocystis pyrifera* and *Saccharina latissima*. *Algal Res.* 26 287–293. 10.1016/j.algal.2017.08.012

[B75] Reed D. C. Carlson C. A. Halewood E. R. Nelson J. C. Harrer S. L. Rassweiler A. (2017). Patterns and controls of reef-scale production of dissolved organic carbon by giant kelp *Macrocystis pyrifera*. *Limnol. Oceanogr.* 60 1996–2008. 10.1002/lno.10154

[B76] Reed D. C. Rassweiler A. Arkema K. K. (2008). Biomass rather than growth rate determines variation in net primary production by giant kelp. *Ecology* 89 2493–2505. 10.1890/07-1106.1 18831171

[B77] Rognes T. Flouri T. Nichols B. Quince C. Mahé F. (2016). VSEARCH: a versatile open source tool for metagenomics. *PeerJ* 4:e2584. 10.7717/peerj.2584 27781170 PMC5075697

[B78] Roth-Schulze A. J. Zozaya-Valdés E. Steinberg P. D. Thomas T. (2016). Partitioning of functional and taxonomic diversity in surface-associated microbial communities. *Environ. Microbiol.* 18 4391–4402. 10.1111/1462-2920.13325 27062175

[B79] Sawabe T. Oda Y. Shiomi Y. Ezura Y. (1995). Alginate degradation by bacteria isolated from the gut of sea urchins and abalones. *Microb. Ecol.* 30 193–202. 10.1007/BF00172574 24185485

[B80] Säwström C. Hyndes G. A. Eyre B. D. Huggett M. J. Fraser M. W. Lavery P. S. (2016). Coastal connectivity and spatial subsidy from a microbial perspective. *Ecol. Evol.* 6 6662–6671. 10.1002/ece3.2408 27777738 PMC5058536

[B81] Schultz-Johansen M. Glaring M. A. Bech P. K. Stougaard P. (2016). Draft genome sequence of a novel marine bacterium, *Paraglaciecola* sp. strain S66, with hydrolytic activity against seaweed polysaccharides. *Genome Announc.* 4 e304–e316. 10.1128/genomeA.00304-16 27103729 PMC4841144

[B82] Sim P. F. Furusawa G. Teh A. H. (2017). Functional and structural studies of a multidomain alginate lyase from *Persicobacter* sp. CCB-QB2. *Sci. Rep.* 7:13656. 10.1038/s41598-017-13288-1 29057942 PMC5651945

[B83] Singh R. P. Reddy C. R. K. (2014). Seaweed–microbial interactions: key functions of seaweed-associated bacteria. *FEMS Microbiol. Ecol.* 88 213–230. 10.1111/1574-6941.12297 24512602

[B84] Stamatakis A. (2014). RAxML version 8: a tool for phylogenetic analysis and post-analysis of large phylogenies. *Bioinformatics* 30 1312–1313. 10.1093/bioinformatics/btu033 24451623 PMC3998144

[B85] Staufenberger T. Thiel V. Wiese J. Imhoff J. F. (2008). Phylogenetic analysis of bacteria associated with Laminaria saccharina. *FEMS Microbiol. Ecol.* 64 65–77. 10.1111/j.1574-6941.2008.00445.x 18328081

[B86] Steneck R. S. Graham M. H. Bourque B. J. Corbett D. Erlandson J. M. Estes J. A. (2002). Kelp forest ecosystems: biodiversity, stability, resilience and future. *Environ. Conserv.* 29 436–459. 10.1017/S0376892902000322

[B87] Tanaka R. Shibata T. Miyake H. Mori T. Tamaru Y. Ueda M. (2017). Temporal fluctuation in the abundance of alginate-degrading bacteria in the gut of abalone *Haliotis gigantea* over 1 year. *Aquac. Res.* 47 2899–2908. 10.1111/are.12740

[B88] Teeling H. Fuchs B. M. Bennke C. M. Krueger K. Chafee M. Kappelmann L. (2016). Recurring patterns in bacterioplankton dynamics during coastal spring algae blooms. *eLife* 5:e11888. 10.7554/eLife.11888 27054497 PMC4829426

[B89] Thomas F. Barbeyron T. Tonon T. Génicot S. Czjzek M. Michel G. (2012). Characterization of the first alginolytic operons in a marine bacterium: from their emergence in marine Flavobacteriia to their independent transfers to marine *Proteobacteria* and human gut *Bacteroides*. *Environ. Microbiol.* 14 2379–2394. 10.1111/j.1462-2920.2012.02751.x 22513138

[B90] Van Tussenbroek B. I. (1989). The life-span and survival of fronds of *Macrocystis pyrifera* (Laminariales, Phaeophyta) in the Falkland Islands. *Br. Phycol. J.* 24 137–141. 10.1080/00071618900650131

[B91] Vollmers J. Frentrup M. Rast P. Jogler C. Kaster A. K. (2017). Untangling genomes of novel Planctomycetal and Verrucomicrobial species from Monterey bay kelp forest metagenomes by refined binning. *Front. Microbiol.* 8:472. 10.3389/fmicb.2017.00472 28424662 PMC5372823

[B92] Wahl M. Goecke F. Labes A. Dobretsov S. Weinberger F. (2012). The second skin: ecological role of epibiotic biofilms on marine organisms. *Front. Microbiol.* 3:292. 10.3389/fmicb.2012.00292 22936927 PMC3425911

[B93] Wang Y. Song Q. Zhang X. H. (2016). Marine microbiological enzymes: studies with multiple strategies and prospects. *Mar. Drugs* 14:171. 10.3390/md14100171 27669268 PMC5082319

[B94] Wargacki A. J. Leonard E. Win M. N. Regitsky D. D. Santos C. N. S. Kim P. B. (2012). An engineered microbial platform for direct biofuel production from brown macroalgae. *Science* 335 308–313. 10.1126/science.1214547 22267807

[B95] Wheeler P. A. North W. J. (1981). Nitrogen supply, tissue composition and frond growth rates for *Macrocystis pyrifera* off the coast of southern California. *Mar. Biol.* 64 59–69. 10.1007/BF00394081

[B96] Whyte J. N. C. Englar J. R. (1978). *Primary Organic Chemical Composition of the Marine Alga Macrocystis Integrifolia Over the Growing Season.* Technical Report, No. 787 Vancouver: Fisheries and Oceans Canada.

[B97] Wong T. Y. Preston L. A. Schiller N. L. (2000). Alginate lyase: review of major sources and enzyme characteristics, structure-function analysis, biological roles, and applications. *Annu. Rev. Microbiol.* 54 289–340. 10.1146/annurev.micro.54.1.289 11018131

[B98] Xu F. Dong F. Wang P. Cao H. Y. Li C. Y. Li P. Y. (2017). Novel molecular insights into the catalytic mechanism of marine bacterial alginate lyase AlyGC from polysaccharide lyase family 6. *J. Biol. Chem.* 292 4457–4468. 10.1074/jbc.M116.766030 28154171 PMC5377765

[B99] Yu G. Smith D. K. Zhu H. Guan Y. Lam T. T. Y. (2017). ggtree: an R package for visualization and annotation of phylogenetic trees with their covariates and other associated data. *Methods in Ecol. Evol.* 8 28–36. 10.1111/2041-210X.12628

[B100] Zhan P. Tang K. Chen X. Yu L. (2017). Complete genome sequence of *Maribacter* sp. T28, a polysaccharide-degrading marine flavobacteria. *J. Biotechnol.* 259 1–5. 10.1016/j.jbiotec.2017.08.009 28811216

[B101] Zhu B. Chen M. Yin H. Du Y. Ning L. (2016). Enzymatic hydrolysis of alginate to produce oligosaccharides by a new purified endo-type alginate lyase. *Mar. Drugs* 14:E108. 10.3390/md14060108 27275826 PMC4926067

[B102] Zhu Y. Thomas F. Larocque R. Li N. Duffieux D. Cladière L. (2017). Genetic analyses unravel the crucial role of a horizontally acquired alginate lyase for brown algal biomass degradation by *Zobellia galactanivorans*. *Environ. Microbiol.* 19 2164–2181. 10.1111/1462-2920.13699 28205313

