# Supplementary material for: Diverse Bacteria Utilize Alginate Within the Microbiome of the Giant Kelp Macrocystis pyrifera
Source: Front Microbiol. 2018 Aug 20;9:1914. doi: 10.3389/fmicb.2018.01914 (PMC6110156; doi:10.3389/fmicb.2018.01914)
Supplement: Supplementary file 4 [file Data_Sheet_1.docx]

Supplemental material for:

**Diverse bacteria utilise alginate within the microbiome of the giant kelp *Macrocystis pyrifera***

Jordan David Lin, Matthew A Lemay, Laura Wegener Parfrey

**Supplemental Figures:**

Figure S1. **Bacterial taxonomy varies substantially between culture-independent and cultured communities**. Taxonomy plots of culture-independent *Macrocystis* (n=44) and water column (n=11) bacterial communities alongside cultured *Macrocystis* (n=18) and water column (n=6) communities. Taxa are displayed at both the level of (**A)** Phylum (*Class for the Proteobacteria) and (**B)** Genus (Family reported if genus was unidentified). The most abundant groups at each level are listed.

Figure S2. **Epibiotic communities differ across *Macrocystis* blade depths.** Bacterial taxa present on the *M. pyrifera* surface are represented at both the level of **(A)** Phylum (*Class for Proteobacteria) and **(B)** Genus (Family reported if genus was unidentified). The most abundant groups at each level are listed. Taxonomic profiles represent *Macrocystis* blades (n=44) from three kelp beds. Blades were sampled at three depths: bottom (1m above sediment), middle (1m below surface), and top (surface of the water column).

Figure S3. **Phylogenetic tree showing distribution of alginate activity within Gammaproteobacteria genera detected in the *Macrocystis* forest.** 16S rRNA sequences from Gammaproteobacteria isolated in this study were placed into a tree of reference sequences using RAxML. Isolates with 99.8-100% identical 16S rRNA sequences were clustered together into operational taxonomic units (OTUs). Green: cultured bacterial isolates in this study; sequences from isolated bacteria with 99.8-100% 16S similarity were collapsed into operational taxonomic units (OTUs). Blue: cultured isolates from this study whose growth was enriched by at least 50% in the presence of alginate, or that grew with alginate as the sole carbon source (marked with *). Red: cultured isolates in this study that degrade alginate. Purple: cultured isolates whose growth was enhanced and that degrade alginate. Gray: cultured isolates demonstrating neither enhanced growth nor degradation. Detailed information for each OTU can be found in Table S1.

Figure S4. **Phylogenetic tree showing distribution of alginate activity within Alphaproteobacteria genera detected in the *Macrocystis* forest**. 16S rRNA sequences from Alphaproteobacteria isolated in this study were placed into a tree of reference sequences using RAxML. Isolates with 99.8-100% identical 16S rRNA sequences were clustered together into operational taxonomic units (OTUs). Green: cultured bacterial isolates in this study; sequences from isolated bacteria with 99.8-100% 16S similarity were collapsed into operational taxonomic units (OTUs). Blue: cultured isolates from this study whose growth was enriched by at least 50% in the presence of alginate. Red: cultured isolates in this study that degrade alginate. Purple: cultured isolates whose growth was enhanced and that degrade alginate. Gray: cultured isolates demonstrating neither enhanced growth nor degradation. Detailed information for each OTU can be found in Table S1.

Figure S5. **Phylogenetic tree showing distribution of alginate activity within Flavobacteriaceae genera detected in the *Macrocystis* forest**. 16S rRNA sequences from Flavobacteriaceae isolated in this study were placed into a tree of reference sequences using RAxML. Isolates with 99.8-100% identical 16S rRNA sequences were clustered together into operational taxonomic units (OTUs). Green: cultured bacterial isolates in this study; sequences from isolated bacteria with 99.8-100% 16S similarity were collapsed into operational taxonomic units (OTUs). Blue: cultured isolates from this study whose growth was enriched by at least 50% in the presence of alginate. Red: cultured isolates in this study that degrade alginate. Purple: cultured isolates whose growth was enhanced and that degrade alginate. Gray: cultured isolates demonstrating neither enhanced growth nor degradation. Detailed information for each OTU can be found in Table S1.

Figure S6. **Comparison of bacterial communities inferred from metagenomic and amplicon datasets.** Taxonomic profiles of bacterial communities from kelp blades at middle (n=4) and bottom (n=5) depths, and from the surrounding water column (n=5), in one kelp bed (Site 3). (**A and B**) Communities reconstructed from metagenomic sequencing at the (**A**) Phylum (*Class for Proteobacteria) level and (**B**) Genus level (black bars indicate genera present in the metagenomic dataset but absent from the amplicon dataset). **(C and D)** Communities reconstructed from amplicon sequencing at the (**C**) Phylum (*Class for Proteobacteria) level and (**D**) Genus level.

**Supplemental Tables:**

Table S1. **Summary of bacterial genera that cultured isolates were assigned to and their reported alginate activity.** Isolates were clustered at 100% 16S rRNA identity into operational taxonomic units (OTUs) and the representative isolate for each OTU is listed.

Table S2. **Summary of differentially enriched bacterial genera and CAZy families between the *Macrocystis* surface and surrounding water column, and across *Macrocystis* blade depths.** Differentially abundant genera were compared across all *Macrocystis* bacterial communities, whereas CAZy families were compared across samples within a single kelp bed (Site 3).

Table S3. **The most abundant CAZy families found within metagenomic functional profiles of *Macrocystis* and water column bacterial communities from a single kelp bed (Site 3).** The top families found within the *Macrocystis* microbiome are reported, along with a brief description of their reported functions. The most abundant CAZy families are also reported for the water column and at different kelp blade depths.

**Supplemental Data Files:**

2. Macrocystis_Bacterial_Community_Metadata.csv

Metadata summary for all kelp and water column bacterial communities (culture-independent and cultured; n=79) from the *Macrocystis* forest that were used in this study.

3. Macrocystis_Bacterial_Isolate_Metadata.csv

Metadata summary for all bacterial isolates that were cultured from the *Macrocystis* surface and surrounding water column in this study.

4. Macrocystis_Bacterial_OTU_Table_SILVA_Raw_Counts.csv

Raw counts of operational taxonomic units (OTUs) present in the *Macrocystis* forest from both culture-independent and cultured bacterial communities found on the *Macrocystis* surface and in the surrounding water column.

5. Macrocystis_Metagenomic_Quality_Statistics.csv

Summary statistics of metagenomic dataset from kelp (n=9; bottom depth=5, middle depth=4) and water column (n=5) bacterial communities within a single kelp bed (Site 3). Quality statistics are from open reading frame annotation using MetaPathways and 16S gene annotation using QIIME.

6. Macrocystis_CAZy2014_Raw_Annotations.csv

Raw counts of annotations from the Carbohydrate Active Enzymes (CAZy) database for kelp (n=9; bottom depth=5, middle depth=4) and water column (n=5) bacterial communities within a single kelp bed (Site 3).

7. Macrocystis_CAZy2014_Bacteria_Annotations.csv

Raw counts of annotations to bacterial genes from the CAZy database for kelp (n=9; bottom depth=5, middle depth=4) and water column (n=5) bacterial communities within a single kelp bed (Site 3).

8. Macrocystis_CAZy2014_Families_Bacteria.csv

Raw counts of bacterial gene annotations mapped to CAZy families for kelp (n=9; bottom depth=5, middle depth=4) and water column (n=5) bacterial communities within a single kelp bed (Site 3).
